# Tape-Worm

**Published:** 1867-08

**Authors:** S. A. McWilliams

**Affiliations:** Chicago, Ill.


ARTICLE XXXV.

### TAPE-WORM. (TÆNIA SOLIUM.)

By S. A. McWILLIAMS, A.M., M.D., Chicago, Ill.

The following case of tape-worm is reported, merely to strengthen the reputed efficacy of pumpkin seeds as an anthelmintic:

Thomas V., a lad of six years, had been troubled for about a year with a tape-worm, segments of which had been frequently seen in his fæces. The parents said that his sleep was often disturbed, as if frightened; that he had occasional pain of a spasmodic character; often picked his nose, and scratched his fundament, and was rapidly wasting away. His appetite was exceedingly variable, sometimes voracious; at others, entirely deficient. Others had subjected the boy to the usual amount of pink root, calomel, castor oil, and turpentine, etc., but the worm safely retained his moorings.

I directed him fifteen drops of turpentine, in the form of an emulsion, three times daily for a week, and afterwards that two ounces of the kernels of pumpkin seeds be thoroughly ground up with sugar, to a very fine pulp, and sufficient mint water added to make an emulsion of twelve fluid ounces.

The above was taken in the morning, between six and seven, in divided doses, upon an empty stomach. At 9 A.M., two tablespoonfuls of castor oil was given, and at 11 A.M., the usurper rapidly beat a retreat before the advancing foe, and twenty-one feet, head and shoulders, were carefully bottled up. Since which time the boy has rapidly gained in strength and spirits to the joy of his doting parents.

